# Pain, power and patience - A narrative study of general practitioners' relations with chronic pain patients

**DOI:** 10.1186/1471-2296-12-31

**Published:** 2011-05-15

**Authors:** Mia Hemborg Kristiansson, Annika Brorsson, Caroline Wachtler, Margareta Troein

**Affiliations:** 1 Lund university, Department of Clinical Sciences, Malmö, Family Medicine, Malmö University Hospital, SE 205 02, Malmö, Sweden

## Abstract

**Background:**

Chronic pain patients are common in general practice. In this study "chronic pain" is defined as diffuse musculoskeletal pain not due to inflammatory diseases or cancer. Effective patient-physician relations improve treatment results. The relationship between doctors and chronic pain patients is often dysfunctional. Consultation training for physicians and medical students can improve the professional ability to build effective relations, but this demands a thorough understanding of the problems in the relation. Several studies have defined the issues that frequently cause problems, but few have described the process. The aim of this study was to understand and illustrate what GPs' experience in contact with chronic pain patients and what works and does not work in these consultations.

**Methods:**

Our theoretical perspective is constructivist, based upon the relativist view that individuals construct realities to understand and navigate the world. Five Swedish General Practitioners (GPs), two male and three female, were interviewed and asked to tell a story about a difficult encounter with a chronic pain patient. Tapes of the interviews were transcribed and analysed using narrative analysis. Three GPs told narratives suited for our analytic tools and these were included in the final results.

**Results:**

Each narrative highlights a certain dilemma and a strategy. The dilemmas were: power game; good intentions that fail when a patient is persuaded against her own conviction; persuasion of the unwilling; transferred tiredness; distrust and dissociation from the patient. Professional strategies of listening, encouraging and teamwork were central to handling difficult situations.

**Conclusions:**

The narratives show that GP's consultations with chronic pain patients sometimes are characterized by conflicts and difficult situations. They are facilitated by methods such as active listening and teamwork, but still may remain hard to handle. This has not before been studied among Swedish GPs. Narratives based on experience are known to be successful in education and this study suggest how narratives can serve as a training of consultation for medical students, but also in Continuing Professional Development groups for experienced doctors in practice.

## Background

Patients with chronic pain are common in general practice [1]. In this paper chronic pain is defined as diffuse musculoskeletal pain associated with neither inflammatory diseases nor cancer. Chronic pain patients are considered a challenge by doctors [1-9]. Suspicion, failure and lack of power characterize doctors' relationships with these patients. Doctors feel suspicious when patients benefit from being ill and when biomedical explanations do not match patients' experience [2-4,6,7]. Doctors fear failure when neither cure, nor improvement nor consolation is achieved. Doctors feel powerless when inadequate resources are paired with problematic life-situations [2-4]. Patients with chronic pain feel questioned and develop different strategies to be perceived as credible [4,5,7,10,11]. The relationship between doctors and patients with chronic pain may often be dysfunctional. Effective patient-physician relations can improve patients' health [2,12,13]. Therefore, negative relationships may have the opposite effect.

Positive effects of consultation training for physicians and medical students have been reported [2,8,14,15]. Appropriate educative changes must however be based on a thorough understanding of problems in the patient doctor relationship, how doctors experience these patients and what happens in consultations with them. Several studies have defined issues that frequently create problems [2-11], but fewer have described in which way the consultation goes wrong.

The aim of this study was to understand and illustrate what GPs' experience in contact with chronic pain patients and what works and does not work in these consultations.

## Methods

### Theoretical approach

In this study our theoretical perspective is constructivist, based upon the relativist view that individuals construct realities to understand and navigate the world. In the constructivist perspective, the role of the researcher is subjective, that is, the researcher is also engaged in construction of reality. Constructivist methodologies are hermeneutic and focus on understanding rather than explaining phenomena [16-18].

In this study we use narrative analysis, in which stories told in interviews are analysed as meaningful entities. Kohler Riessman points out: "it is well suited to studies of subjectivity and identity" [18]. Narrative studies are useful in education since they are often "memorable, grounded in experience and encourage reflection"[19]. Narrative analysis differs from other qualitative research by investigating the story as whole rather than thematic processing across several interviews [18]. Our definition of narrative is primarily based on the work of Labov [18,20,21]. We use "narrative" and "story" synonymously, "listeners" are the researchers and all readers of the study. In Labov's definition a narrative tells about an explicit past event, is built up of universal components following a chronological sequence. The components are: *abstract *(a summing up of the story); *orientation *(time, place, situation, participants); *complicating action *(course of events); *evaluation *(importance and meaning of the event and the narrator's attitude); *resolution *(what happened eventually); *coda *(goes back to present). Our definition is broader, inspired by Riessman. It includes Labov's components, but also considers that narrative can be a part of a text about an event, or series of events, with a beginning and end [18]. We also omit Labov's chronology.

This study has also been inspired by James Gee [22]. For Gee, the way things are told is the most important indicator of intended meaning. This gives access to what often is neglected by other methods and what intuitively often feels important for an interviewer while doing the transcriptions, for example pauses, tone of voice, laughter and other elements. Labov's and Gee's structural methods complement each other. In Gee's analysis the way of telling divides speech into stanzas, defined as "a group of lines about a single topic; each stanzas captures a single *"vignette" */---/ they are often four lines long" [22]. Gee suggests a linguistic analysis including observation of the use of pronouns, adverbs etc. Using Gee we have divided the narratives into stanzas, and focused on some linguistic aspects.

### Data collection

All informants were general practitioners (GPs). This speciality was chosen because GPs often have long-term relationships with chronic pain patients. In Sweden doctors can certify sickness insurance benefits which are publically financed through income tax. At most health care centres patients can register with one specific family doctor.

Informants were chosen to represent different ages, sexes, workplaces, and length of experience. One informant was in specialist training and the most experienced had been a GP for about 30 years. Three were female and two male and they worked at rural and urban health care centres in Sweden. The main criterion for selection was the expected willingness and capability of sharing stories about chronic pain patients. MHK and CW had no prior knowledge of the informants. Narrative method focuses on individual experience [20], and here five informants were estimated to be sufficient.

Three months before the interviews in 2008 we sent potential informants a letter with a presentation and a request for participation. All accepted. The GPs were asked to prepare stories about chronic pain patients. They were named Dr A- Dr E in the order they were interviewed. Dr A was interviewed in 2005 as the first part of an undergraduate thesis; the study was continued in 2008 with interviews with Drs B-E. MHK conducted all interviews in Swedish, the first language for her and the informants. The interviews were recorded and lasted about an hour each. Informants were initially all asked one question: Can you tell me about a patient with chronic pain, or a consultation with such a patient, that has evoked strong reactions from you? Follow-up questions encouraged story development.

### Analysis

MHK transcribed all recorded material verbatim including questions, pauses and underlined stressed words. MHK and AB conducted an analysis together. CW did a parallel analysis. The two analyses turned out to be very similar. MHK did a second transcription based on stories strictly about chronic pain patients, in total twelve narratives. These contained Labov's narrative components but did not follow his chronology [21]. For each doctor we selected the richest narrative according to Labov's criteria. The interviews with Dr D and Dr E did not contain narratives as defined by Labov. These interviews contained generalizations about patient-physician relations, based on encounters that happened long ago. Therefore, they were not included in the final analysis.

A third transcription inspired by Gee was performed on the three remaining narratives by re-listening to the tapes [20]. They were divided into stanzas and given vignettes, descriptions of the major topic of the lines [22]. We paid close attention to length of stanzas, pauses and sighs, pronouns, repetition of words, synonyms and stressed words. Each narrative was structurally and thematically explored case by case.

The citations were translated after analysis. In the final transcription *descriptions of scenes, pauses and questions etc. are written in italics*. Stressed words are underlined, and *words or ways of telling worth commenting written in italics and underlined*. These signs: /---/ indicate that a fragment of the stanza has been excluded.

## Results

We found that each narrative illustrated a particular dilemma and a strategy to deal with chronic pain patients.

### Dr A

Dr A was middle-aged male who had worked as a GP for more than 10 years. He told a story about a woman who, despite her long term problem with diffuse pain, mainly had been working. He told about several phone calls and exhausting consultations about a disagreement over a sickness certificate. A pain rehabilitation clinic later supported half-time sickness absence but the relationship remained strained.

The narrative is fragmented and the narrator often moves from descriptions of scenes to generalizations, especially when describing something negatively charged. The narrative shows a power game between doctor and patient in which the doctor has the upper hand. The following consultation occurred after a telephone conflict:

Stanza 28 - distressed, offended, and disappointed

...a few weeks later so, so she came very *distressed *and I think *she was crying *in the waiting room already and, and she had *really prepared for the meeting *and really wanted to tell me how *I had offended *her and how *disappointed *she was with me and...

Stanza 29 - receive a dressing-down

*I *had to sit here in ten minutes and sort of *receive *what she had to say.

Strong words are used to portray her. He is passive and has to "*receive"*. Dr A describes this as part of a "chess game". She thinks he offended her on the telephone, and he now steps back while she moves forward. We note the ping-pong effect of the repeated use of "she", "I":

Stanza 73 - want to examine before a doctor's letter

"Well, in the conversation today I said so: - O.k. *you want *me to refer you for an X-ray, eeh *then I want *to examine you first. You'll get an appointment before lunch tomorrow."

Stanza74 -"good" and hang up

-Good she said and we hung up.

They negotiate. The opposition of the pronouns "she" and "I" underline lack of mutual understanding. Eventually they agree and he expresses a rare "we" with his patient. Otherwise "we" in this narrative refers to colleagues. Negotiation is not between equals, however. Throughout the narrative it is the doctor who defines the terms of engagement. For example, Dr A hesitates about renewing a sickness certificate initiated by another doctor:

Stanza 51 - not obvious without own assessment

/---/ Oh then I said that it wasn't all that obvious that I should do it...eeh, naturally not without having made my *own assessment *and maybe not even on *principle */---/

He stresses his professional duty to make his own assessment. His reluctance "*on principle" *expresses power. At the end of the interview he explicitly states how difficult it is to have power over people's finances, a major part of their conflict.

The informant stresses how demanding these consultations are, but he also describes how he handles them:

Stanza 129 - prepare for a race

*Eeh...*so then I try to stick to my method, not to let, to see to it that I have a good time before, this is as I said a demanding meeting. Sort of *like an athlete prepares himself for a race*, you don't go there the day after a big party and *without having tied your shoes *and so on, but you *make sure your equipment is ok*, you go and pee first, you have like....maybe switched on this lamp as we have done /*the lamp showing if occupied or not*/, and you make sure that there are good preconditions so you have a good *chance*...*to make it*.

Dr A's methods of preparing, staying calm and listening attentively are also ways of maintaining control of the circumstances of the consultation. The narrative shows conflict, strained negotiations, and power imbalance, but at the end she is still his patient, and his method seems to work.

### Dr B

Doctor B was a younger woman, halfway through specialist training. When they met, her middle-aged female patient had been on sickness absence for more than a year with pain related to cervicobrachial syndrome. Initially Dr B prolonged her sickness certificate. The trouble started when she wanted her patient back to work.

The central dilemma is about an unwilling patient who is persuaded against her own will and despite her gain from a sick-role. The result is disappointment for patient and doctor, conflicts with other professionals, and a new sick leave. It is also a story where the doctor's good intentions and methods fail. The narrative gradually reveals an uncertainty in the description of their relationship. However, it first declares Dr B's intention to create a platform for their relationship, and why she initially prolonged her patient's sick leave:

Stanza 4 - build a safe relationship

/---/ and the first time I met here I felt that, no I can't just make a break here, but in some ways I felt that we had to *build *some kind of form of contact so she can like feel *safe *with me and feel that what I say is like the best for the patient.

The narrative contains components of a good doctor-patient relation. Dr B allies with her patient by using "we". However, a distance between doctor and patient is created by referring to her as "the patient". Shortly this distance is increased by use of the pronoun "I", a figure that has the power to consider if the patient's sickness absence should be ended or not.

Stanza 7 - see how it goes

But then *we *had yet the time before decided that: Next time *I *think probably that *we *will finish this and see like...how it goes.

"I" is now used consistently and the narrator tries to persuade her patient:

Stanza 9 - very high risk

But still I didn't feel convinced that it was *the absolute best for the patient*. And I had said to her, in fact already from the beginning that if you don't start to work when you have been on sick leave as long as this there's a great risk that you...said that *the risk is that you'll never work again, and it is really high*. Ooh...

One responsibility of the doctor is to give advice based on medical knowledge. In stanza 9 this is done in an affirmative and persuasive way. Gradually the description displays uncertainty and doubt about the patient and their relationship. Already in stanza 4 where Dr B declares her good intentions, the word "like" is interwoven. The rest of the narrative is permeated by small hesitant words:"like","probably","yet". She recalls what happened when she suggested ending the sick leave.

Stanza 11 - wants to try

Eeeh...and...when we finally had discussed it I felt *yet *that, well I did get the patient on my side *quite *well. She wasn't completely satisfied, but she said that: no but I still want to try, I don't want to be on sick leave for the rest of my life and/---/, then I said let us try.

The stanza tells us that the persuasion was successful, but at the same time the hesitant words undermine this success. Note again the use of "I" and "she" instead of "we". Dr B is initially satisfied:

Stanza 13 - helped her

Yes an' that felt good at the time. It did, it felt really good then, I felt that I had really helped to do the patient a service and I had like, I had like....helped her.

Dr B is convinced that she has acted according to professional standards, and that her patient has been helped. But soon the patient seeks conflict through the medical social worker.

Stanza 14 - she was really sad

But then some days later she called our medical social worker who she had contact with and she was really sad about this and *felt *that she *didn'tatall want *like to be unemployed /---/

The distrust is obvious and in Dr B's narrative the question is not the need for sick leave, but a question of the patient's preference for sickness absence before unemployment. Dr B does not however give in to these demands. Dr B has not seen this patient since, partly due to training on another clinic. She expresses frustration that another doctor has given the patient a new sickness certificate. One of the basic professional intentions, to help people, failed:

Stanza 18 - no help

Oh I thought in what way does that help the patient?.../---/

Eventually she shares one thing with her patient - their mutual disappointment:

Stanza 19 - she disappointed

But I get the feeling that she, she probably doesn't want to see me again, I do think so. She was probably...*disappointed *that it ended as it did, I think so...mmm/*pause*/

The struggle to build a stable relationship and change the patient's life comes to a dead-end in frustration and disappointment. The professional method gives rise to backlash and the unwilling patient manages to stay in her sick-role through another doctor.

### Dr C

Dr C was in her sixties and had been a GP for about three decades. Her narrative was about an obese middle-aged woman with diabetes and possibly fibromyalgia. She was a former patient of two of Dr C's colleagues, but had now registered with Dr C. She wanted a prolonged sickness certificate, which her previous doctor had denied her. Dr C tried to judge whether this was a reasonable request, but decided that they needed another meeting to sort things out. Their first meeting was the day before this interview.

This narrative is about the overwhelming feeling of getting a patient's whole life on your lap. The patient is described as unbelievably tired and dysphoric, exhausted even by doing the washing up. The narrative is told in a tired voice, with sighs and long pauses; the stanzas are generally short. This transfers tiredness, disbelief, and dissociation from the patient to the listener. The consequential use of "she" and "I" emphasize this distance, and the use of "we" is reserved for cooperation with the medical social worker. This reflects Dr C's major method of dealing with these patients: teamwork with other categories of professionals.

After their first consultation Dr C argues with her colleague:

Stanza 8 - scolded

/---/ First I went out and scolded the other doctor who had had her [the patient] listed for a long time and who should have written this certificate that now was *placed on my lap */*coughs*/

She expresses anger but also a sense of that something has been dumped on her:

Stanza 9 - her whole tiredness over me

/*small laugh in the voice*/ Oh when I came home yesterday, I have never been so tired, so I got somehow *her whole tiredness over me*...I think that was what happened. /*short pause*/I was completely finished...

Dr C is not in control. The patient's choice of Dr C as her GP is out of her control, as is the emotional drain on Dr C after meeting the patient. Before these stanzas words expressing tiredness were used five times in a couple of minutes, here emphasised with "never". However, Dr C's colleague offers to write the sickness certificate anyway, but is stopped by Dr C:

Stanza 11 - will be challenged

**/---/ **I feel a bit challenged by these kind of patients too. And...eh..I thought that if she now has decided that I should be her doctor and then I have to meet that challenge even if it can be really hard, and even if I can't help her at all!/---/...oh eh /*sighing*/

This stanza shows for respect for the patient's choice and interest in grasping the reasons for a patients' life situation. But the description of the patient contradicts this:

Stanza 26 - while away one's days

/---/...and how she *whiled away her time *I never clearly understood so that was no good ....[meeting]...

Stanza 26 phrase implies that the patient is not using her time for anything meaningful at all. The description of this woman is illustrative:

Stanza 38 - sad and still

This is a short woman, but she weighs 118 kilos or something like that so *she is totally square and she sat there like a big lump and was perfectly still and looked . . . /short pause/ dejected*.

She even reflects that in this case she might not want to know the whole story anyway. It all boils down to a conclusion:

Stanza 33 - a person's whole life

/---/...oh, it isn't easy to do your [job], you have a half hour and you sit there, with a person's whole life /*quiet again and a little laughter*/ oh it's not possible, it is a pretty . . . pretty impossible assignment really, and still, it is what we are supposed to do /*smacking her lips/ *mmm.

A doctor's duty is difficult when a person's whole life is the problem causing the pain.

Stanza 52- life hurts

/---/ It is possible that she has fibromyalgia, or else it's just life that hurts . . . /*pause*/ ah /*sigh*/ and that, that I don't know that much about . . . /*laugh*/

Several times we find laughs as in stanza 52. Dr C acknowledges that some cases are impossible and distances herself from the patient as well as from her own professional standards. Later in the narrative she seeks support from other health care professionals. Keeping some distance and asking for support are Dr C's methods for handling the difficult situation

## Discussion

### Summary of main findings

The narratives illustrate how GPs handle patients with chronic pain. Each narrative highlights a certain dilemma and a strategy. Dr A's narrative illustrates a power game about a sick-leave certificate; Dr B's story demonstrates persuasion of the unwilling; Dr C's account transfers tiredness and expresses distrust of and dissociation from the patient. All three narratives show that an intrinsic part of the doctor's role in these dilemmas is the power inequality between doctor and patient. Listening, encouraging, keeping distance and teamwork are the informants' professional tools to handle these difficult situations.

### Strengths and limitations

Narrative analysis is a methodology that provides a good starting point for exploring research questions. Narrative analysis is a form of case study and as such is appropriate to use when initially exploring a research area, as in our case, as well as to provide a base for theoretical inference and future research. Compared with other qualitative methodologies it maintains high resolution of informants' experience of events, allowing for close observation and preventing loss of detail [18]. In this study we chose narrative analysis because we wanted to catch the dynamics of challenging encounters, in order to examine specific examples of how the consultation can go wrong.

Narrative studies often present one case, even though in some reports several interviews have been collected [20]. The stories told by Dr A, Dr B and Dr C all had strong illustrations. By keeping three narratives in the final analysis we demonstrate breadth of doctor experience. Equally important, the informants overlap on issues such as conflict about sick leave and distrust of the patient due the possible benefit of a sick role. This does not make general conclusions possible, but functions as a qualitative validation that these are important issues.

Validation in qualitative research is more appropriately addressed as trustworthiness [20]. This study fulfilled several criteria for trustworthiness within narrative methodology suggested by Riessman [20]: an explicitly described methodology; use of tape-recorder; verbatim transcriptions; use of theoretical ideas from Labov and Gee; consideration of coherence of the narratives and concordance of the parallel analytic interpretations.

An open interview results in lengthy and varied material. We found that freshness of experience gave the narratives emotional immediacy. All experiences for every human have multiple perceptions and the context and narrator's intention influences the version that is presented [23]. Maybe these fresh experiences were not filed away into the person's professional role yet, which could explain their immediacy with fewer tendencies to abstraction and generalization. The initial interview question could have been narrowed and formulated differently to capture only narratives about recent experiences. Maybe partly due to the formulation of the question, two doctors (Dr D, Dr E) mainly generalized instead of telling coherent stories about tangible cases. However we also believe that their telling depended on this being a difficult subject to talk about and that one of them had had little clinical practice recently before the interview took place.

### Comparison with existing literature

There are several similarities between this study and other research about relations between physicians and chronic pain patients. Conflict and strained relations are often pointed out [3,5,7,9,24]. Kenny showed that at worst neither the doctor nor the patient act as listeners, but both as speakers. Both sides can distance themselves by placing the other in an unfavorable group [7]. All doctors in our study struggle to increase their listening and overcome dissociation from their patients. Only when they succeed in this does the relationship stabilize, with Dr A as an obvious example.

A common source of conflict is the origin of pain; biological according to the patient, and psychosomatic according to the doctor. This is illuminated by Dr A and Dr C in their distrust and unwillingness to write a sickness certificate. All the narratives demonstrate the problem of having influence over the patient's private economy [7,11].

The struggle for power is obvious in the narratives of Dr A and Dr B. Power issues within the patient-physician relation have been studied [3,5,7,25]. Goodyear-Smith notes that the best way of empowering both the doctor and the patient is that both acknowledge the power issues and behave respectfully. She also claims that when a doctor cannot cure a disease, healing may be facilitated by letting patients tell their life-stories [25]. Dr B acts according to this, but her effort to be medically correct makes her force a decision against the patient's will. The sense of powerlessness seen in Dr C's narrative is frequently described in other research [2,6,7].

Various attempts to improve the relationship between chronic pain patients and their physicians are explored in numerous studies. Other studies have shown that biomedical advances lead to better physician-patient relationships, for example through better understanding of disturbed pain modulation, new pharmacological approaches, or through physiotherapy and cognitive behaviour therapy [24,26]. Increased knowledge of biological mechanisms reduces distrust, and new treatments augment the possibilities available for the doctor. Awareness of an increased risk of psychopathology among pain patients at pain clinics can also help the doctor [27].

Empowerment is an umbrella term for various approaches aimed at increasing patient influence, sometimes called shared decision making [8,28-30]. Empowerment means using communication strategies to appreciate the experience of the patient and redistribute power to her [4,28,29]. Dr C can be said to demonstrate empowerment. By aiming to grasp the reasons for her patient's life-situation and by realizing her own limits as a doctor Dr C makes room for and gives responsibility to her patient. Respected family physicians in USA sometimes use empowerment without calling it by name [9]. The keys to their successful consultations are collaboration, appropriate use of power, and empathy. Empathy accompanied by listening is stressed in other studies as well [3,4,2]. All our informants seek to fulfil these aims and succeed to some extent, but their narratives also reveal how challenging it can be to implement these keys in clinical practice with patients perceived as difficult.

## Conclusions

This study points at the difficulties physicians experience with patients they cannot cure or efficiently relieve from pain. The results emphasize consultation skills as important tools in these situations, but even with the best of methods, experienced physicians may encounter severe problems in their relations with chronic pain patients.

Narratives based on experience work well in education [19]. The results of this study can be applied in training of consultation skills for medical students, but also in Continuing Professional Development groups (CPD) for experienced doctors in practice.

## Abbreviations

GP: General Practitioner; CPD: Continuing Professional Development

## Competing interests

The authors declare that they have no competing interests.

## Authors' contributions

MHK, MD, has been a radio journalist and has a BA in literature. CW is a MD, house officer, PhD, and has a BA in anthropology. AB is MD, PhD and specialist in general practice. MT is MD, PhD, senior lecturer and specialist in general practice. Both have extensive experience of qualitative research and studies of patient-physician relationships. Our backgrounds influence our use of the narrative method. MT was responsible for the major planning of the study. MHK performed the interviews. MHK, AB, and CW performed the analysis. All researchers contributed to the writing process. All authors read and approved the final manuscript.

## Pre-publication history

The pre-publication history for this paper can be accessed here:

http://www.biomedcentral.com/1471-2296/12/31/prepub
